# Carotid DSA based CFD simulation in assessing the patient with asymptomatic carotid stenosis: a preliminary study

**DOI:** 10.1186/s12938-018-0465-9

**Published:** 2018-03-12

**Authors:** Dong Zhang, Pengcheng Xu, Hongyu Qiao, Xin Liu, Liangping Luo, Wenhua Huang, Heye Zhang, Changzheng Shi

**Affiliations:** 1 Department of Medical Imaging Center, The First Affiliated Hospital, Jinan University, No. 613, Huangpu Road West, Tianhe District, Guangzhou, 510630 Guangdong Province China; 2 Department of Anatomy, Guangdong Provincial Key Laboratory of Medical Biomechanics, School of Basic Medical Science, Southern Medical University, Guangzhou, 510515 China; 3 Institute of Advanced Computing and Digital Engineering, Shenzhen Institutes of Advanced Technology, 1068 Xueyuan Ave, Xili University Town, Nanshan, Shenzhen, 518055 Guangdong Province China

**Keywords:** CFD, Carotid stenosis, Perfusion, Angiography

## Abstract

**Background:**

Cerebrovascular events are frequently associated with hemodynamic disturbance caused by internal carotid artery (ICA) stenosis. It is challenging to determine the ischemia-related carotid stenosis during the intervention only using digital subtracted angiography (DSA). Inspired by the performance of well-established FFRct technique in hemodynamic assessment of significant coronary stenosis, we introduced a pressure-based carotid arterial functional assessment (CAFA) index generated from computational fluid dynamic (CFD) simulation in DSA data, and investigated its feasibility in the assessment of hemodynamic disturbance preliminarily using pressure-wired measurement and arterial spin labeling (ASL) MRI as references.

**Methods:**

The cerebral multi-delay multi-parametric ASL-MRI and carotid DSA including trans-stenotic pressure-wired measurement were implemented on a 65-year-old man with asymptomatic unilateral (left) ICA stenosis. A CFD simulation using simplified boundary condition was performed in DSA data to calculate the CAFA index. The cerebral blood flow (CBF) and arterial transit time (ATT) of ICA territories were acquired.

**Results:**

CFD simulation showed good correlation (r = 0.839, *P* = 0.001) with slight systematic overestimation (mean difference − 0.007, standard deviation 0.017) compared with pressure-wired measurement. No significant difference was observed between them (*P* = 0.09). Though the narrowing degree of in the involved ICA was about 70%, the simulated and measured CAFA (0.942/0.937) revealed a functionally nonsignificant stenosis which was also verified by a compensatory final CBF (fronto-temporal/fronto-parietal region: 51.58/45.62 ml/100 g/min) and slightly prolonged ATT (1.23/1.4 s) in the involved territories, together with a normal left–right percentage difference (2.1–8.85%).

**Conclusions:**

The DSA based CFD simulation showed good consistence with invasive approach and could be used as a cost-saving and efficient way to study the relationship between hemodynamic disorder caused by ICA stenosis and subsequent perfusion variations in brain. Further research should focus on the role of noninvasive pressure-based CAFA in screening asymptomatic ischemia-causing carotid stenosis.

## Background

Internal carotid stenosis is the main cause of hemodynamic disturbance and the subsequent stroke in brain [1]. Furthermore, asymptomatic internal carotid stenosis affects approximately 7% of women and more than 12% men older than 70 years, and most of them have the stenosis ≥ 50% [2]. However, most of the patients are not aware of this condition until fatal events happen. The management of patients with asymptomatic internal carotid stenosis is challenging in clinical practice because the definition of the high-risk patients with severe asymptomatic carotid stenosis remains unclear [3–5].

At present, angiography is the clinical standard to evaluate the severity of carotid stenosis, including Doppler ultrasonography, computed tomography angiography (CTA), magnetic resonance angiography (MRA), and digital subtraction angiography (DSA) [6–8]. However, these routine methods can only detect the anatomical carotid stenosis. According to Kasner’s research, the probability of stroke is 0.08 in 1 year to the patients with stenosis 50–69%, and is 0.23 to the patients with stenosis ≥ 70% [6]. The alteration of hemodynamic function plays an important role in assessing the risk of stroke to patients with asymptomatic internal carotid stenosis [9, 10].

The fraction flow reserve (FFR) has been well-established in evaluating the hemodynamic characteristics of coronary artery stenosis [11]. Using a pressure-sensing guidewire, Han and Liu have validated the feasibility of pressure gradient measurement in intracranial large arteries and vertebral artery in clinic, respectively [12, 13]. However, guidelines for the application of pressure wire in carotid arteries has not yet been established, and the high cost and invasive practice may be the main reasons [14, 15]. The advance of computational fluid dynamic (CFD) could be an alternative and efficient tool to make up for its shortages, and the noninvasive FFR measurement based on coronary CTA data have presented good performance in diagnosing the ischemic lesions [16, 17]. Furthermore, hemodynamic characteristics and flow patterns on the site of carotid stenosis can also be calculated and visualized by combining the contrast-enhancement MR angiography (CE-MRA) or DSA with CFD [18–20]. But few focused on the trans-stenotic pressure gradient measurement of internal carotid artery, and its relationship with risk of ischemic stroke in brain still lacks enough evidence [21].

Perfusion is an important parameter to estimate dysfunction in the level of brain tissues [22, 23]. The compromised cerebral blood flow (CBF) caused by internal carotid stenosis indicates a risk for future ischemic stroke in brain [9, 24]. Arterial spin labeling (ASL) has emerged as a useful tool for CBF evaluation in clinical practice because of its noninvasive, nonradioactive and nonpoisonous advantages [25–27]. Furthermore, ASL is highly susceptible to the arterial transit time (ATT) which is associated with collateral blood flow [28], and it would be ideal to apply ASL with multiple post-labeling delay (PLD) time to estimate the CBF and ATT simultaneously [29].

In this study, based on a case with asymptomatic unilateral internal carotid stenosis, we introduced a pressure-based carotid arterial functional assessment (CAFA) index generated from CFD simulation in DSA data, and further investigated its feasibility in the assessment of hemodynamic disturbance preliminarily using invasive pressure-wired measurement and multi-delay multi-parametric ASL-MRI as references.

## Methods

This study was conducted in accordance with the principles of the Declaration of Helsinki and met the requirement of medical ethics. The Local Ethical Review Committee approved this research. Since this study was retrospective in nature, the informed consent was waived and anonymized data was used for analysis.

### Case presentation

A 65-year-old man with a history of hypertension for 11 years and type 2 diabetes mellitus (T2DM) for 3 years treated with amlodipine and perindopril once a day and insulin injection every day. His hypertension and T2DM were considered to be controlled. He was recommended a Doppler ultrasonography of cervical arteries as a screening due to his vascular risk factors. Doppler ultrasonography showed an atherosclerotic stenosis at the proximal site of left internal carotid artery (ICA) which was characterized as homogeneous plaque. There was a peak systolic velocity increase of 168 cm/s at the site of stenosis. The morphology data presented a carotid stenosis of 69%. And then, DSA were performed and the results confirmed a severe stenosis at the proximal site of left ICA, and the degree of narrowing was also estimated about 70% according to the NASCET criteria (North American Symptomatic Carotid Endarterectomy Trial). A pressure-sensing guidewire was used when the invasive carotid angiography was performed. Besides, brain MRI revealed no abnormal lesions. For further evaluation, a pseudo-continuous arterial spin labeling (pCASL) MRI data with multiple post-labeling delay (PLD) was acquired in this case.

### MRI protocols and data processing

MRI examination was performed on a 3.0 T MR system (GE Healthcare Discovery MR 750, Waukesha, WI) with 8 channel head coils. The pCASL scans were performed using background suppressed and a stack-of-spirals 3D-fast-spin echo imaging sequence with the following parameters: PLD = 1.0/2.0/3.0 s; TR/TE, 4658/11.1 ms; FOV, 240 × 240 mm; matrix, 512 × 8; section thickness, 3.0 mm; bandwidth 62.5 kHz and NEX, 3.

CBF maps at each single PLD were obtained offline using an independent workstation (Advantage Workstation 4.5, GE Healthcare) based on the following equations:

1 $${\text{WD}} = \left[ {\mathop \sum \limits_{i = 1}^{4} w\left( i \right)\Delta M\left( i \right)} \right]/\left[ {\mathop \sum \limits_{i = 1}^{4} \Delta M\left( i \right)} \right]$$

where $\Delta M\left( i \right)$ is the mean perfusion difference images for each PLD, and $w\left( i \right)$ is the PLD (1.0/2.0/3.0 s). A weighted delay ${\text{WD}}$ is calculated by Eq. (1) and converted into ATT or $\delta$ based on the theoretical relationship between ${\text{WD}}$ and ATT [27]. CBF at each delay,${\text{f}}\left( {\text{i}} \right)$, is calculated using the measured ATT map and the Eq. (2).

2 $$f\left( i \right) = \frac{{\lambda \Delta MiR_{1a} }}{{2\alpha M_{0} \left[ {\exp \left( {\left( {\hbox{min} \left( {\delta - w\left( i \right),0} \right) - \delta } \right)R_{1a} } \right) - \exp \left( { - \left( {\tau + w\left( i \right)} \right)R_{1a} } \right)} \right]}}$$

where $R_{1a}$(= 0.72/0.61/s at 1.5/3 T) is the longitudinal relaxation rate of blood, $M_{0}$ is the equilibrium magnetization of brain tissue, $\alpha$ (= 0.8) is the tagging efficiency, $\tau$ (= 1.5 s) is the duration of labeling pulse, $w\left( i \right)$ is the PLD (1.0/2.0/3.0 s), $\lambda$ (= 0.9 g/ml) is the blood/tissue water partition coefficient. The final CBF is defined as the mean of estimated CBF at each PLD [30].

### Digital subtraction angiography and pressure-wired measurement

The 3D-DSA examination of cerebral and carotid arteries was carried out with a rotational angiographic system (Artis zeego, Siemens Healthcare, Forchheim, Germany) and was done with nonionic contrast medium (Visipaque, GE Healthcare) 2–2.4 ml/s with a total injection volume of 15–18 ml. The 3D-DSA was performed with a C-arm rotation of 180°.

According to the protocol introduced by Han [12], the reference blood pressure (mPa) was acquired at the cervical portion of internal carotid artery (C1) by the catheter, and then a pressure wire (PressureWire Aeris/Certus, St. Jude Medical, St. Paul, USA) was positioned distal to the stenosis of interest (at least 3 cm downstream of the lesion) and the mean distal arterial pressure (mPd) was measured. Finally, the guidewire was pulled back, and the pressure gradient was record continuously. The invasive CAFA was calculated by dividing the mPd by mPa.

### CFD configuration

#### Boundary condition

The volumetric flow rate was derived using the lumen volume divided by the transport time. One can easily confirm two frames when the blood flew through the inlet of the reconstructed vessels. Based on DSA data set, we could obtain the flux as well as the lumen volume during two frames. The volumetric flow rate divided by area of the inlet was defined as the inlet boundary. As for the outlets, outflow conditions were applied.

The blood motion was governed by Navier–Stokes equations as

3 $$\uprho\left( {\frac{\text{du}}{\text{dt}}\, + \,{\text{u}} \cdot \nabla {\text{u}}} \right)\, = \, - \,\nabla {\text{p}}\, + \,\upmu\nabla^{\text{2}} {\text{u}}\, + \,{\text{f}}$$

4 $$- \nabla \cdot {\text{u}} = 0$$

We assumed that blood can be described as incompressible Newtonian fluid with a density of 1046 kg/m^3^ and a viscosity of 0.04 dynes/cm^2^. The wall of the blood vessels supposed to be rigid without slipping.

#### Noninvasive CAFA measurement

Fractional flow reserve was an important indicator for ischemia of the carotid artery. Carotid arterial functional assessment (CAFA) index was derived from the concept used in coronary arteries and was defined as the ratio of flow in the stenotic vessel to the flow in the same vessel without stenosis.

$${\text{CAFA}}_{v} = \frac{{{\text{Q}}_{\text{H}}^{\text{S}} }}{{{\text{Q}}_{\text{H}}^{\text{N}} }}$$

where ${\text{Q}}_{\text{H}}^{\text{S}}$ is the flow rate at the stenotic vessel, ${\text{Q}}_{\text{H}}^{\text{N}}$ is the flow rate at the same normal vessel. When applied to the suspected stenotic arteries, the normal artery model can be determined as the arteries with stenosis removed. According to the theory proposed by Pijls et al. the CAFA may have an approximate simplified expression based on directly measured data [11].

$${\text{CAFA}}_{p} = \frac{{{\text{mP}}_{\text{d}} }}{{{\text{mP}}_{\text{a}} }}$$

#### Statistical methods

Eighteen points were selected through the CFD simulation and pressure wire derived pull-back curves respectively, and the Pd/Pa was calculated one to one. The paired t-test, Pearson correlation and Bland–Altman plots were used to assess the accordance between the CFD simulation and invasive measurements. A P-value less than 0.05 was deemed statistically significant. All the analyses were performed on the SPSS (version 14, Chicago, IL, USA) and MedCalc Software (MedCalc, Mariakerke, Belgium).

## Results

**Fig. 1:**
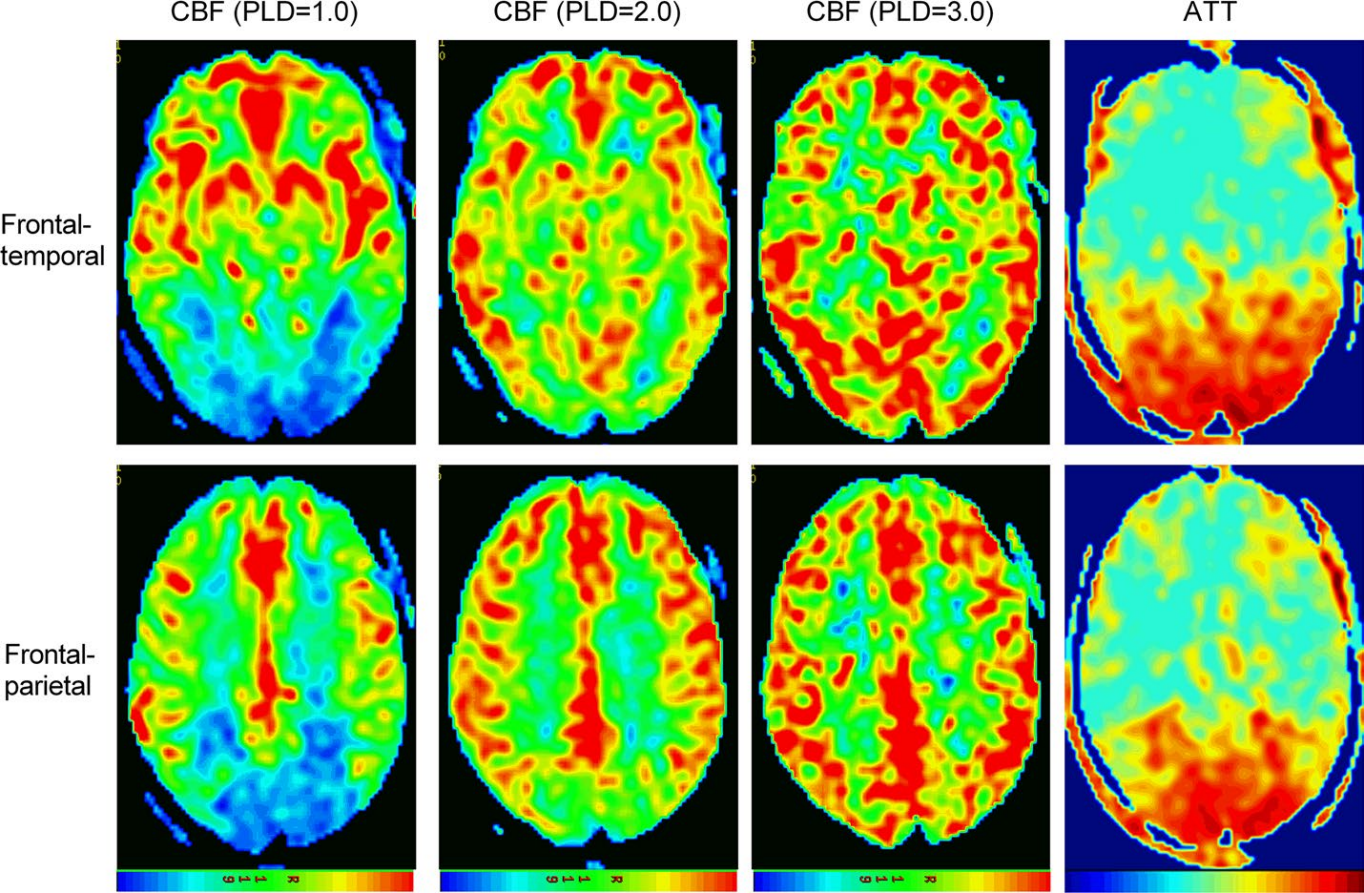
The parametric CBF and ATT maps of presented case. CBF with 1.0 PLD decreased slightly in the involved left ICA territories compared with the contralateral hemisphere, especially in the left fronto-parietal. With the increasing of PLD, CBF increased in the involved territories and was close to the right hemisphere in spite of a slightly prolonged ATT in left

The perfusion data of left and right ICA territories measured by the pCASL was presented at Table 1 and Fig. 1. Compared with the contralateral hemisphere, CBF with 1.0 PLD decreased slightly in the involved left ICA territories, especially in the left fronto-parietal. With the increasing of PLD, CBF increased in the involved territories and the final CBF was close to the right hemisphere in spite of a slightly prolonged ATT in left.

**Table 1:** The results of CBF (ml/100 g/min) and ATT (s) in ICA territories

	Fronto-temporal			Fronto-parietal		
	Left (involved)	Right (normal)	Δ%	Left (involved)	Right (normal)	Δ%
CBF (PLD = 1.0 s,)	51.76	52.93	− 2.21	32.54	36.98	− 12.0
CBF (PLD = 2.0 s)	50.61	49.73	1.77	49.55	49.78	− 0.46
CBF (PLD = 3.0 s)	52.38	48.02	9.08	54.76	53.05	3.22
Final CBF	51.58	50.23	2.69	45.62	46.6	2.10
ATT	1.23	1.13	8.85	1.40	1.31	6.87

*CBF* cerebral blood flow, *PLD* post-labeling delay, *ATT* arterial transit time, *ICA* internal carotid artery

Δ% was left–right percentage difference

**Fig. 2:**
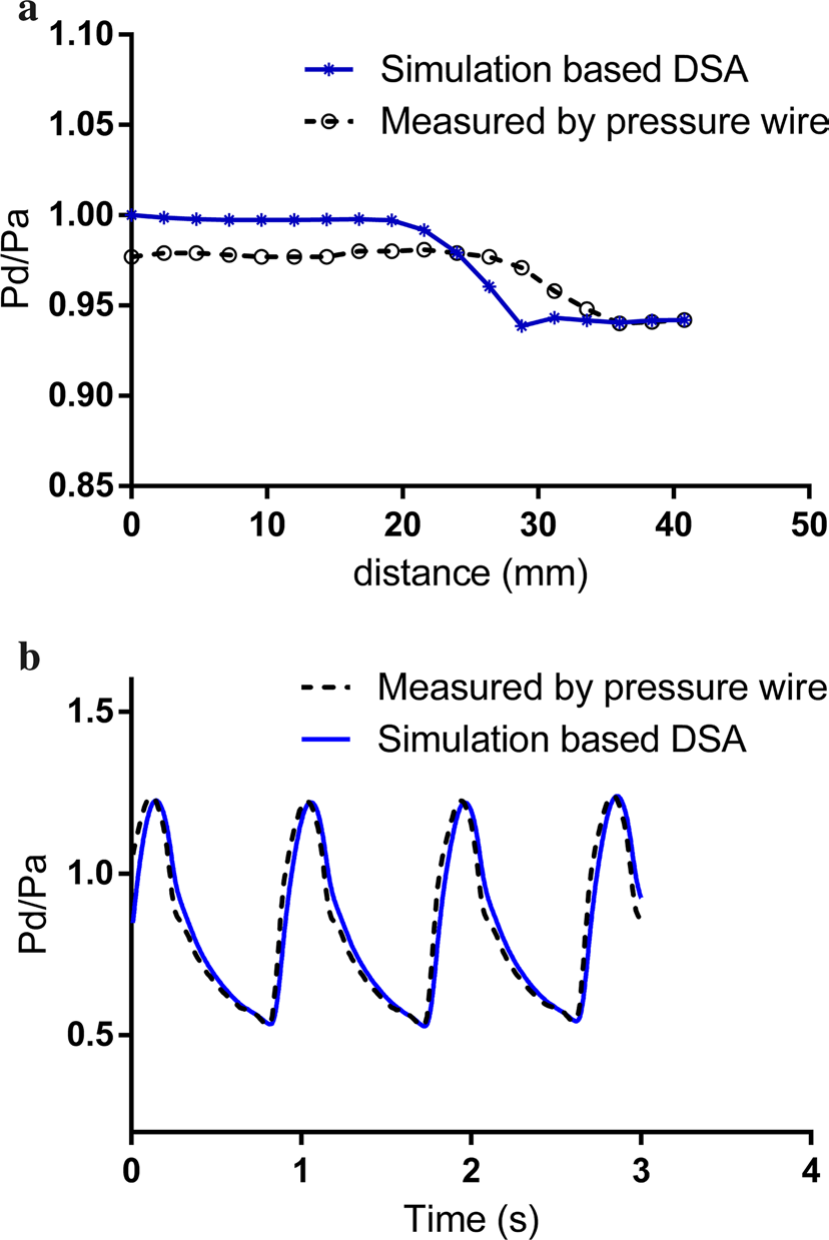
Pressure profiles calculated with CFD simulation and measured by pressure wire. **a** Pressure ratio versus distance along the centerline. **b** Pressure ratio versus time at the boundary

As showed in Fig. 2a, the pressure ratio (Pd/Pa) calculated from DSA data set was presented, as well as the ones measured by pressure wire; and two curves both appeared a sharpen decrease around 20 mm, where the stenosis is. Finally, the invasive CAFA measured by guide-wire was 0.937, and the noninvasive CAFA was calculated as 0.942 within 14 s using CFD simulation.

**Fig. 3:**
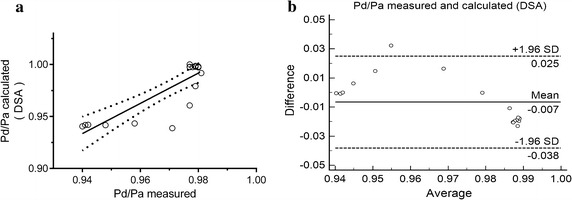
Comparison of pressure gradient ratio (Pd/Pa) between CFD simulation and invasive measurement. Pearson correlation analysis **a** and Bland–Altman plots **b** of calculated Pd/Pa based DSA data sets compared with the measured result; r was 0.839 with significant difference (P = 0.001), and mean difference was − 0.007 with standard deviation 0.017

Pressure profile obtained by invasive way was presented as comparison of the simulation. As showed in Fig. 2b, the pressure curves calculated by simulation and measured by pressure guide-wire were presented respectively, and were overlapped well. Furthermore, the simulation results and the pressure-wired measurements showed good agreement (r = 0.839, P = 0.001, Fig. 3a), and no significant difference was observed between two methods (P = 0.09). Additionally, the Bland–Altman plot presented a slight systematic overestimation of calculated Pd/Pa based DSA (mean difference − 0.007, standard deviation 0.017, Fig. 3b).

**Fig. 4:**
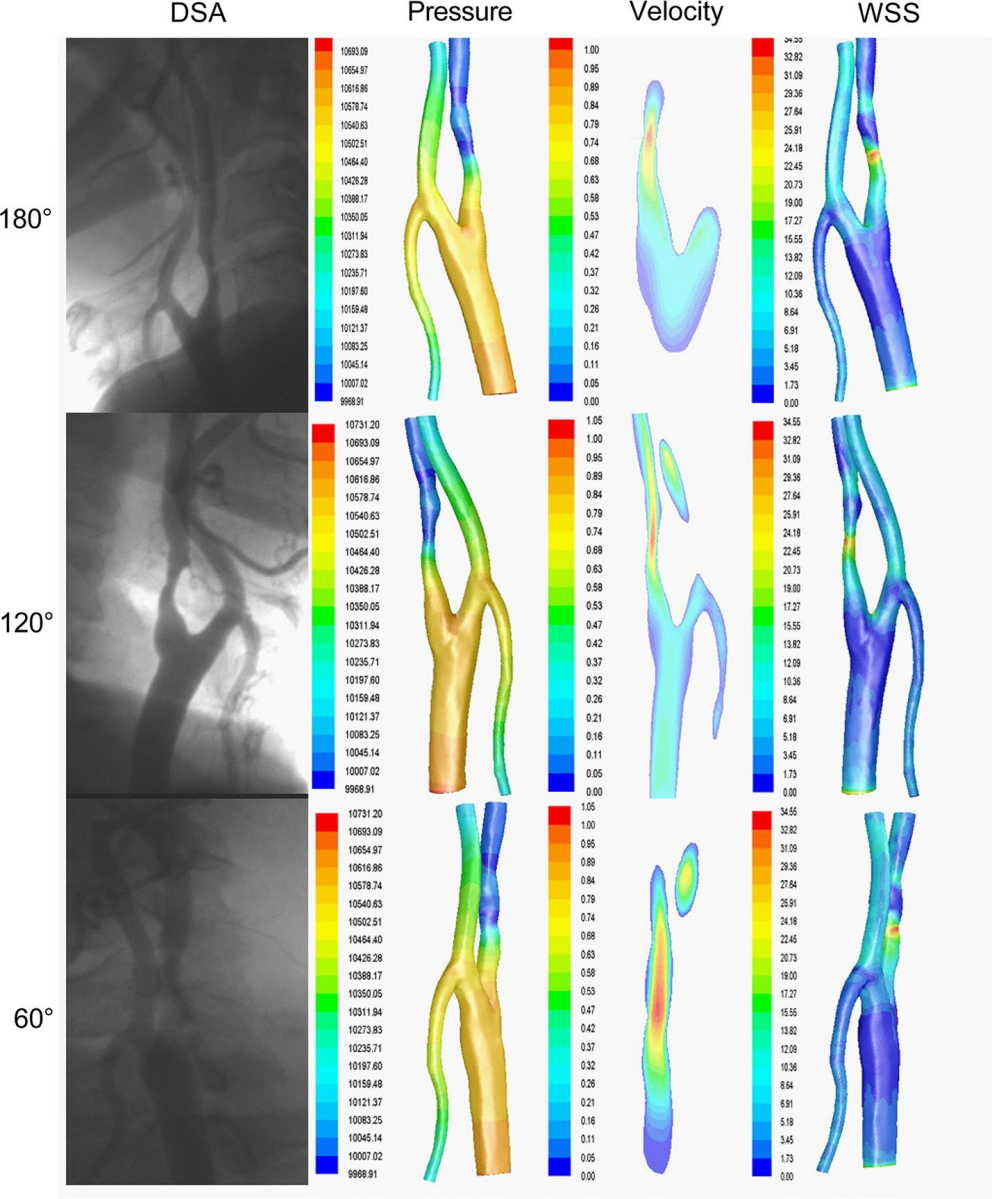
The hemodynamic distributions of carotid artery simulated with CFD at three typical angles. At the site of stenosis, the pressure decreased nearly 400 Pa while the velocity increased nearly 0.5 m/s, and the wall shear stress (WSS) increased nearly 30 Pa

Furthermore, we also depicted a set of hemodynamics distributions of the carotid artery with three typical angles (0°, 60°, 120°) in Fig. 4. The hemodynamic distributions varied with the decrease of lumen radius. At the site of stenosis, the pressure decreased nearly 400 Pa while the velocity increased nearly 0.5 m/s; as for the wall shear stress (WSS), it increased nearly 30 Pa.

## Discussion

The treatment for patients with asymptomatic carotid artery stenosis is highly controversial in clinic because of lacking the direct and quantitative indicators to assess the risk of stroke in brain [3, 31, 32]. Hemodynamic analysis plays an important role in making decision before the artery revascularization [9, 10]. In this case, we performed pCASL MRI with multiple PLD times to evaluate the hemodynamic characteristics in brain together with an invasive pressure gradient measurement of carotid stenosis and an introduced CFD method based on the DSA data.

Our results showed that CBF of the involved left ICA territories at 1.0 PLD decreased compared with the contralateral hemisphere, especially in the left fronto-parietal. However, with the increasing of PLD, CBF increased in the involved left ICA territories, and the final CBF was close to the right hemisphere in spite of a slightly prolonged ATT in left. The compromised cerebral blood flow (CBF) caused by internal carotid stenosis indicates a risk for future ischemic stroke in brain [9, 24]. Most ASL studies employ a single post-labeling delay (PLD) time between 1.5 and 2.0 s to compute the CBF [26]. However, ASL is highly susceptible to the arterial transit time (ATT) [28]. When a prolonged ATT caused by arterial stenosis and collateral blood flow is greater than the employed PLD, the CBF will be underestimated, and it would be ideal to apply ASL with multiple PLDs to estimate the CBF and ATT simultaneously [29]. In previous perfusion studies on carotid stenosis, a “misery perfusion” was defined as CBF <35 ml/100 g/min or ATT> 7.89 s, and normal left–right difference were known to vary ± 10% [33, 34]. Finally, based our results of ASL, we did not think this patient has the high risk of ischemic stroke even though the degree of narrowing is severe; and the absence of intraplaque hemorrhage and lipid-rich necrotic core reduce the risk of rupture. Finally, we excluded the choice of carotid stenting or endarterectomy, and treated him with intensive medical therapy adding aspirin, clopidogrel, diuretic, and statin.

Invasive functional assessment based on pressure gradient has been proved to be applicable in internal carotid artery quantitatively [12]. In the presented case, even though the left carotid stenosis was severe (estimated about 70%), the acquired invasive CAFA was 0.973. According to Han’s research, revascularization was performed in indicated lesion only if the pressure gradient ratio was 0.7 or less which had been widely used in coronary stenosis [12]. While Han also suggested that the measured mPd/mPa in carotid artery is not equal to the FFR in coronary due to the lack of induced hyperemia [12]. But Liu argued that the resistance of cerebral and neck vascular is low and constant which should be neglected, and the induced hyperemia is not necessary [13]. However, pressure-based carotid functional assessment could not be widely used to indicate the management in the clinic, because it lacks enough evidence to identify the diagnostic criterion. Multi-delay multi-parametric pCASL may be an effective way to provide a direct and quantitative evidence to identify the diagnostic standard of CAFA.

The high cost of pressure wire and the invasive medical operation may hinder the application of hemodynamic measurement in clinic [14]. As an alternative method, we introduced a CFD simulations based on DSA data in this study, and calculated the CAFA to help diagnose the ischemia-related carotid artery stenosis. Our results showed a good accuracy between pressure simulation and pressure-wired measurement. Furthermore, using the velocity derived from DSA data to simplify the computational model, we obtained the reliable results within 15 s which made it prepared for the fast analysis in clinic. Previous studies of CFD simulation have focused on coronary artery to assess the hemodynamic characteristics, and fast computation of pressure ratio from coronary angiography, acquired with or without pharmacological hyperemia-induction, is feasible [17, 35]. In our study, the calculated CAFA is 0.942 based DSA data. According to Liu’s study on the functional assessment of stenotic carotid artery by CFD-based pressure gradient evaluation, the value of 0.88 was suggested as an indicator to classify the severe and mild-to-moderate stenosis [21]. However, in Liu’s research, the severity of carotid stenosis was categorized based on peak systolic velocity (PSV) and morphology measured by ultrasound, and it neglected the compensation of collateral circulation and lack direct evidence to identify the ischemia-related carotid stenosis quantitatively. Therefore, with its advantage in saving costs, reducing procedure time and lowering invasive risk, it is necessary to investigate the potential of CFD-based CAFA in screening the ischemia-related carotid artery stenosis, and use the advanced multi-delay multi-parametric pCASL-MRI as a reference.

Even though the selected points showed good agreement between simulation results and the pressure-wired measurements, there was a constant difference between the CFD and in vivo results in Fig. 2a, as well as an outlier in Fig. 3b. As the pressure recording is only related to the time and speed when the pressure sensor was pulled back, and the pull-back curves was estimated according to the recording data, it is difficult to realize the absolutely precise fitting during the data processing. Besides, these phenomena may also be caused by the limited resolution of the carotid DSA, especially the region proximal to the stenosis, which may lead to the deviation between reconstructed geometries and in vivo anatomy. Furthermore, only the cross-sectional area was used to calculate the average flow of internal carotid artery, the volume of the simulation domain was not taken into the consideration which may also cause the error. Further adjustment taking a correction of the actual vascular volume into the simulation domain is required, and the improvement of the image quality is also necessary to reduce the deviation caused by geometric effects.

As a preliminary study, we restricted to only one typical patient’s imaging data sets to verify the method of CFD simulation in carotid stenosis. Actually more cases were necessary for a full validation, and a single center study including normal and ischemia cases is needed in the future. Besides, the measurement of CAFA we implemented was according to Han’s introduced procedure [12], and further research on the establishment of standard protocol is necessary.

## Conclusions

Despite these limits, our study introduced a noninvasive method to evaluate the hemodynamic disturbance of asymptomatic carotid artery stenosis quantitatively. Based on the DSA data, we simplified the CFD simulation and calculated the pressure-based CAFA index efficiently. The calculated pressure gradient ratio showed a good consistence with the pressure-wired measurement, and the noninvasive CAFA index revealed a functionally nonsignificant stenosis in a 65-year-old man with severe asymptomatic unilateral ICA stenosis, which can be verified by cerebral multi-delay multi-parametric ASL-MRI. Therefore, the DSA based simulation has the potential to be used in studying the relationship between hemodynamic disorder in ICA stenosis and subsequent perfusion variations instead of costly pressure wire. Our current research provided a possibility of noninvasive pressure-based CAFA in screening asymptomatic ischemia-causing carotid stenosis. Further research including normal and ischemia cases should been put on the agenda.

## Abbreviations

ICA: internal carotid artery
DSA: digital subtracted angiography
CAFA: carotid arterial functional assessment
CFD: computational fluid dynamic
pCASL: pseudo-continuous arterial spin labeling
CBF: cerebral blood flow
ATT: arterial transit time
CTA: computed tomography angiography
FFR: fraction flow reserve
CE-MRA: contrast-enhancement MR angiography
PLD: post-labeling delay
T2DM: type 2 diabetes mellitus
WSS: wall shear stress
